# Prevention of incisional hernia using different suture materials for closing the abdominal wall: a comparison of PDS, Vicryl and Prolene in a rat model

**DOI:** 10.1007/s10029-019-01941-9

**Published:** 2019-05-20

**Authors:** S. van Steensel, L. C. L. van den Hil, A. Bloemen, M. J. Gijbels, S. O. Breukink, J. Melenhorst, K. Lenaerts, N. D. Bouvy

**Affiliations:** 1 Department of General Surgery, Maastricht University Medical Centre, P. O. Box 5800, 6202 AZ Maastricht, The Netherlands; 2 NUTRIM School of Nutrition and Translational Research in Metabolism, Maastricht University, Maastricht, The Netherlands; 3 Department of General Surgery, VieCurie Medical Centre, Venlo, The Netherlands; 4 Department of Pathology, Maastricht University Medical Centre, Maastricht, The Netherlands

**Keywords:** Incisional hernia, Prevention, Suture, PDS, Vicryl, Prolene, Rat model, Abdominal wall

## Abstract

**Purpose:**

An incisional hernia occurs frequently after a midline incision with an incidence of 12.8%. The choice in suture material used for abdominal wall closure is not straightforward and the conflicting literature focuses on clinical outcomes. This study compares a non-absorbable, slow-absorbable and fast-absorbable suture in a rat model, focusing on histological outcomes predicting better fascia healing.

**Methods:**

33 male Wistar rats, divided over three groups, each received two separate 1 cm incisions closed with either Prolene 4/0, PDS 4/0 or Vicryl 4/0. At 7 days and 21 days, one of the incisions was explanted. Tissue was semi-quantitatively scored regarding inflammatory cells and collagen fibres present. Using qPCR macrophage polarisation, fibroblast activity and vascularisation were evaluated. Data were analysed by Kruskal–Wallis test with Mann–Whitney *U* post hoc test. A *p* value of 0.017 was considered significant after Bonferroni correction.

**Results:**

All animals recovered without complications and completed the 21 days of follow-up. The Vicryl group showed a higher presence of macrophages after 21 days in comparison with Prolene (*p* = 0.003) and PDS (*p* = 0.006) and more foreign body giant cells compared to Prolene at 7 days (*p* = 0.010) and PDS at 21 days (*p* < 0.001). qPCR showed 2.5-fold higher expression of clec10A in PDS compared to Prolene after 7 days (*p* = 0.007).

**Conclusions:**

The results of this study carefully support the use of PDS suture, compared to Prolene and Vicryl, in abdominal wall closure based on a favourable macrophage response. The heterogeneity and variability in the data might be explained by the spectrum of the macrophage subtype paradigm.

**Electronic supplementary material:**

The online version of this article (10.1007/s10029-019-01941-9) contains supplementary material, which is available to authorized users.

## Introduction

An incisional hernia is a frequent complication following abdominal surgery and is defined as any abdominal wall gap with or without a bulge in the area of a postoperative scar perceptible or palpable by clinical examination or imaging [1, 2]. Bulging, discomfort or pain and cosmetic issues have a significant impact on the quality of life of patients [3].

At a weighted mean follow-up of 23.7 months, 12.8% of the patients have developed an incisional hernia and the estimated risk to undergo an incisional hernia repair after a midline incision is 5.2% [4]. In high-risk patients, the incidence increases to up to 30% [5, 6]. The identified patient-related risk factors are obesity (BMI > 25 kg/m^2^), the presence of an abdominal aortic aneurysm and congenital connective tissue disorders [7–12]. Furthermore, postoperative wound complications are an independent risk factor for the development of an incisional hernia [9].

With unacceptable high recurrence rates after incisional hernia repair, which ranges from 23.8% to 32% [13–16], a preventive strategy has become the focus of scientific research. In high-risk patients, preventive mesh placement proves an effective tool in reducing incisional hernia rates [6, 17, 18]. However, if the mesh is placed intra-peritoneally, delayed wound healing and increased pain after 6 week follow-up are observed [19]. Thus, improving the technique of primary abdominal wall closure might be advantageous.

The technique for closure of the abdominal wall after a midline incision has evolved from using interrupted sutures to a continuous running monofilament suture technique [20, 21]. Also, a small stitch suture technique, with a 4:1 suture length to wound length ratio, decreases the incidence of incisional hernia [22, 23]. The European Hernia Society formulated a guideline for the closure of the abdominal wall incorporating the previous statements. This guideline makes a weak recommendation for abdominal wall closure with slow-absorbable sutures, which is adopted in standard clinical practice [24]. Multiple meta-analyses report conflicting results regarding the preferable suture material, caused by substantial heterogeneity between studies and inclusion criteria [4, 20, 21, 25].

Two meta-analyses suggested the use of slow-absorbable sutures based on a lower occurrence of suture sinuses and wound pain, but in the absence of a difference in incisional hernia occurrence [21, 25]. The most recent meta-analysis recommended slow-absorbable sutures based on a decrease of incisional hernia compared to fast-absorbable sutures, but a separate comparison between only slow-absorbable and non-absorbable sutures was not performed [20]. Lastly, Bosanquet et al. suggested in a meta-regression that suture material does not have influence on incisional hernia rate or on the occurrence of suture sinuses [4].

Collagen metabolism plays a central role in the healing of the abdominal wall. Collagen maturation and collagen breakdown in particular are crucial. A decreased collagen I/III ratio is indicative of a low presence of mature collagen. Also increased MMP activity, responsible for collagen type I denaturation, is involved in abdominal hernia development [26]. However, the healing process of the abdominal wall is still not fully understood. This unknown factor complicates the discussion regarding incisional hernia prevention. The aim of this study is to investigate the effect of different types of suture materials on the healing of the abdominal wall in a rat model. Giving insight to physiological and pathophysiological processes might lead to new starting points for preventive strategies.

## Materials and methods

The protocol was approved by the animal ethics committee of the Maastricht University conform the Dutch Experiments on Animals Act.

## Materials

A non-absorbable monofilament polypropylene suture (Prolene 4/0, Ethicon Inc; Johnson & Johnson, Somerville, NJ, USA), a slow-absorbable monofilament polydioxanone suture (PDS II 4/0, Ethicon Inc; Johnson & Johnson, Somerville, NJ, USA), and a fast-absorbable multifilament polyglactine suture (Vicryl 4/0, Ethicon Inc; Johnson & Johnson, Somerville, NJ, USA) were obtained commercially.

### Study design

33 male Wistar rats between 240 and 260 g were acquired from a registered breeding facility (Envigo, Horst, the Netherlands), housed at the Maastricht University animal facilities and cared for according to local protocol. The animals were socially housed in filter-topped cages with a 12 h day-night cycle and had free access to food and water. Because of hormonal influences on wound healing by progesterone and oestrogen [27] and a faster postoperative weight recovery of males compared to females [28], only male rats were used in this experiment. The animals were randomly assigned to one of the three groups for abdominal wall closure of two separate midline incisions by either Prolene 4/0, PDS 4/0 or Vicryl 4/0. There were two time points for tissue evaluation, after 7 days and 21 days of follow-up.

### Procedure

After a 1-week acclimatisation period, preoperative pain medication (buprenorphine 0.05 mg/kg) was administered. Anaesthesia was induced using 3–4% isoflurane and maintained with 2.5% isoflurane through inhalation of an air mixture. The animal was placed on a sterile field after the abdomen was shaved and the skin was disinfected with 2% iodine solution.

Via a midline incision of 6 cm in the skin, the abdominal wall was exposed. Two smaller full-thickness incisions of approximately 1 cm were made in the midline of the exposed abdominal wall, with a minimal distance of 2 cm between the two incisions. The two incisions were closed with either Prolene 4/0, PDS 4/0 or Vicryl 4/0 using a continuous suture technique; the skin was closed with Monocryl 4/0 (Ethicon Inc; Johnson & Johnson, Somerville, NJ, USA). Postoperatively, the animals were administrated fluid resuscitation and recovered under a heat lamp.

After 7 days of follow-up, one of the closed midline incisions was explanted and after 21 days of follow-up the second previously closed midline incision was explanted after killing, using the operative procedure as described above. Using the previous midline incision, randomly either the upper or lower abdominal wall incision was explanted equally distributed over the experimental groups. The explanted specimens were cut in half and one-half was preserved in liquid nitrogen and the other half in formaldehyde 4% for further analysis. The animals were killed by carbon dioxide overdose at the completion of the follow-up at 21 days.

### Histology

Half of the explanted incision was fixed in 4% formaldehyde, dehydrated and embedded in paraffin. Subsequently, 4 µm thick tissue sections were cut and a haematoxylin–eosin staining was performed. An experienced animal pathologist, who was blinded to the group allocation, evaluated the stained sections microscopically. To compare inflammation and collagen deposition, granulocytes, macrophages, foreign body giant cells and collagen fibres were scored using a four-point semi-quantitative scoring system (not present, slightly present, moderately present or abundantly present) [29–32].

### RNA isolation and quantitative real time PCR

Total RNA was isolated from the snap-frozen abdominal wall specimen using TRI reagent (Sigma, NL). 750 ng DNAse-treated RNA was used to synthesise cDNA (SensiFAST™, cDNA synthesis kit, Bioline, London, UK). For qPCRs, a volume of 10 µl consisting of the cDNA equivalent of 2.5 ng total RNA, 1 × Absolute qPCR SYBR Green Fluorescein Mix (SensiFAST™ SYBR^®^ Hi-ROX Kit, Bioline, London, UK) and 0.15 μM of gene-specific primers (Sigma, NL) was used (Supplementary Table 1). The LightCycler^®^ 480 Instrument II (Roche Molecular Systems, Inc., was used to perform the qPCR. LinRegPCR software was used to establish gene expression levels. The geometric mean of two internal control genes, Rplp0 and beta-actin (Actb), was calculated and used as normalisation factor. In one sample in the Vicryl group, insufficient cDNA was available for analysis with Actb, resulting in unreliable data. Rplp0 showed no expression in one sample from the Prolene group. For both samples, one reliable housekeeping gene was available, which was used as normalisation factor. Relevant primers were identified from the literature and build using a primer designing tool (Primer-blast) [33]; the sequences are reported in Table 1.

**Table 1:** All primers were tested for transcription of the intended gene

Gene name	Product length		GC %	Sequence
*rplp0* (ribosomal protein lateral stalk subunit P0)	190	f	55.00	CCTCACCGAGATTAGGGACA
		r	45.00	ATCGCTCAGGATTTCAATGG
*actb* (actin, beta)	297	f	55.00	CCGCGAGTACAACCTTCTTG
		r	55.00	CAGTTGGTGACAATGCCGTG
*il6* (Interleukin 6)	246	f	57.14	CTCTCCGCAAGAGACTTCCAG
		r	47.62	TTCTGACAGTGCATCATCGCT
*nos2* (iNOS)	234	f	52.38	TAGTCAACTACAAGCCCCACG
		r	60	GTGAGGAACTGGGGGAAACC
*cd86* (CD86)	164	f	45.45	AGACATGTGTAACCTGCACCAT
		r	55	TACGAGCTCACTCGGGCTTA
*il10* (Interleukin 10)	186	f	52.38	CGACGCTGTCATCGATTTCTC
		r	60.00	CAGTAGATGCCGGGTGGTTC
*clec10a* (C-type lectin domain containing 10a)	164	f	60.00	GAGGCTTGAGCCAGAAGGTG
		r	52.38	TGCTGAGCCGTTGTTCTTGAG
*mrc1* (mannose receptor C typ 1)	212	f	60.00	CCCGCTCCTCAAGACAATCC
		r	55.00	AAATACGGTGACTGCCCACC
*cd163* (CD163)	131	f	60	CTCTGAAGCGACGACAGACC
		r	50	ATGCCAACCCGAGGATTTCA
*tgfb1* (transforming growth factor-β)	115	f	60.00	GGCTGAACCAAGGAGACGGA
		r	55.00	CCTCGACGTTTGGGACTGAT
*vegfa* (vascular endothelial growth factor a)	235	f	60	AGAAGGGGAGCAGAAAGCCC
		r	47.83	GATCCGCATGATCTGCATAGTGA
*angpt2* (angiopoietin 2)	168	f	55	CATGATGTCATCGCCCGACT
		r	52.38	TCCATGTCACAGTAGGCCTTG
*nos3* (eNOS)	139	f	52.38	GAATGGAGAGAGCTTTGCAGC
		r	60	CCGCCAAGAGGATACCAGTG
*col1a1* (collagen type 1 alpha 1 chain)	237	f	60	CTGACTGGAAGAGCGGAGAG
		r	55.00	CAGGATCGGAACCTTCGCTT
*mmp1* (matrix metallopeptidase 1)	144	f	55.00	AAGGCCACTGGTGATCTTGC
		r	43.48	GGTATTTCCAGACTGTTTCCACA
*fn1* (Fibronectin 1)	165	f	63.16	TCCCCTCCCAGAGAAGTGG
		r	43.48	TTGGGGAAGCTCATCTGTCTTTT

### Statistical analysis

A sample-size calculation was performed in preparation of the experiment. A difference of 20% in inflammation on a histological level was considered relevant, with a variance of ± 16%. Alpha was chosen at 0.05 and with a power of 0.80, resulting in a needed group size of 11 animals per group.

All data were expressed as a median with range or mean with 95% confidence interval. Nonparametric tests were performed using a Kruskal–Wallis test. In case of significance, a Mann–Whitney *U* post hoc test was performed to identify specific differences between the groups. A Bonferroni correction was used to correct for multiple testing, so a *p* value of 0.017 (0.05/3) was considered significant. SPSS 23.0 for Mac (SPSS Inc., Chicago, IL, USA) was used for the statistical analysis.

## Results

Recovery was uncomplicated for all rats. No postoperative complications were encountered, and all animals completed the 21 days of follow-up.

### Histology

For the histological evaluation, 31 out of 33 samples at 7 days and 30 out of 33 samples at 21 days were available for analysis. Two samples in the PDS group were missing for both time points and one sample for the Vicryl group at 21 days of follow-up.

**Fig. 1:**
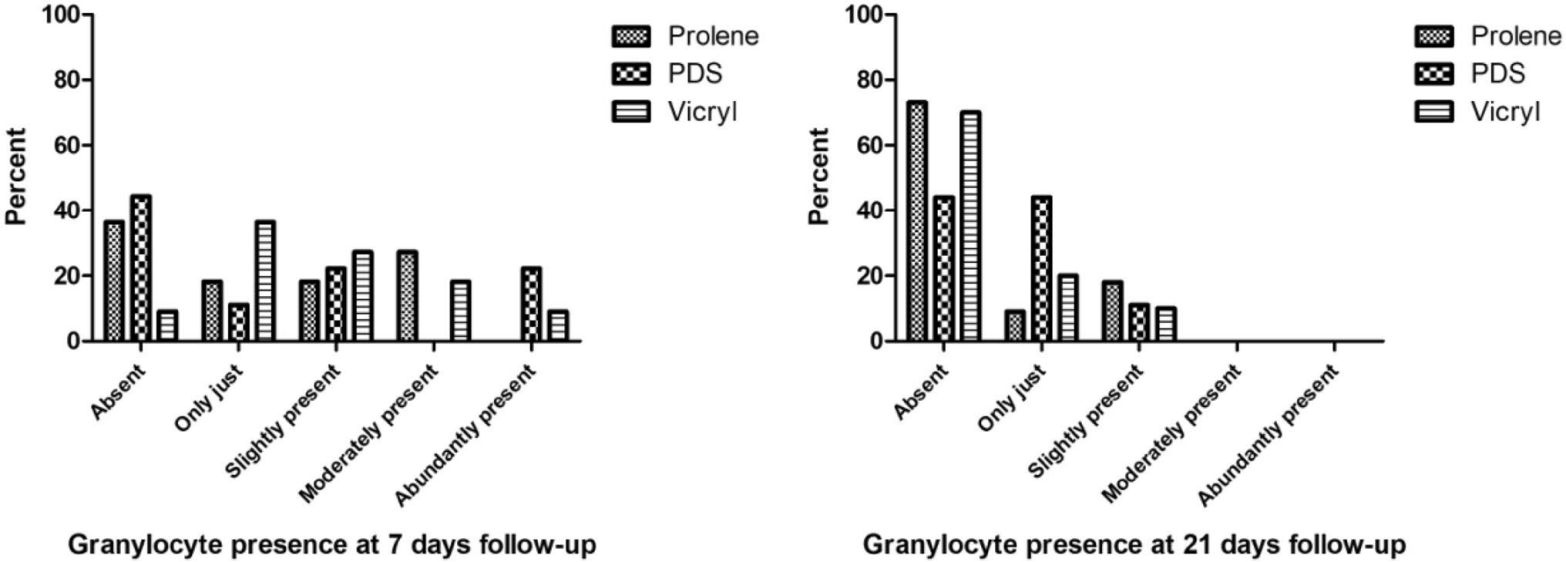
Semi-quantitative scoring of the presence of granulocytes at 7 days and 21 days follow-up per suture type. At 7 days: *p* = 0.621, at 21 days: *p* = 0.539

**Fig. 2:**
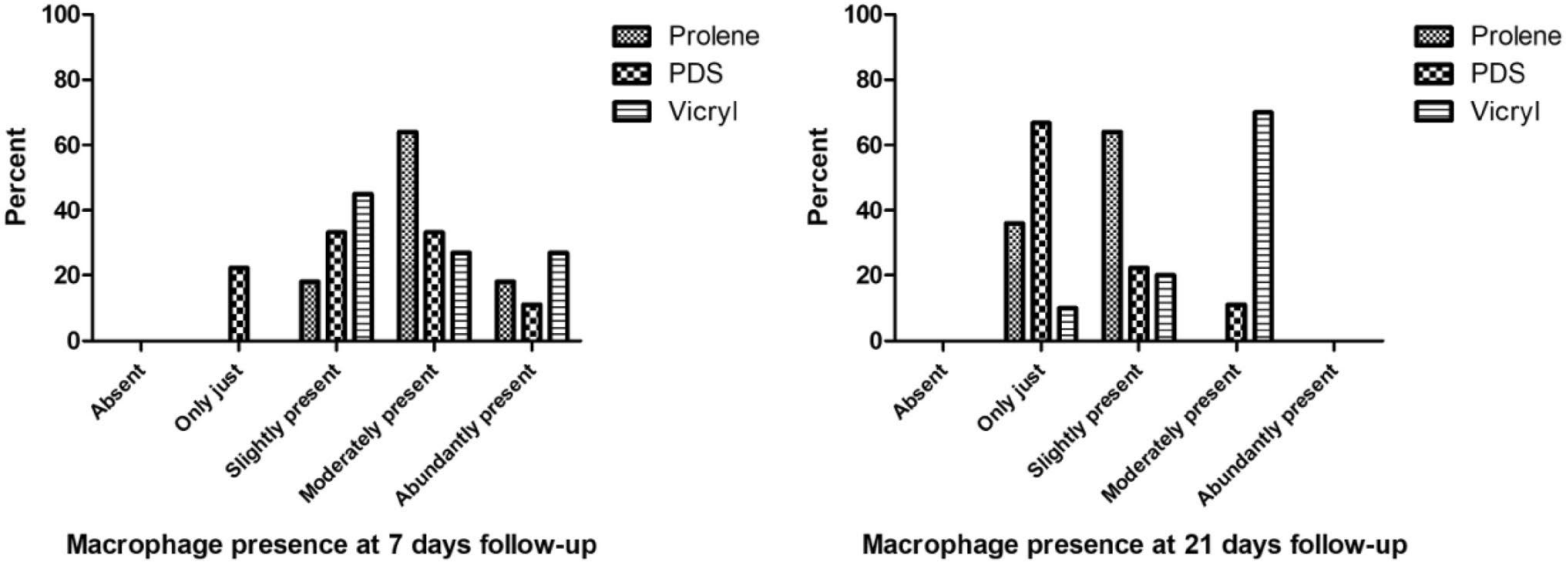
Semi-quantitative scoring of the presence of macrophages at 7 days and 21 days follow-up per suture type. At 7 days: *p* = 0.257, at 21 days: Prolene vs Vicryl *p* = 0.006, PDS vs Vicryl *p* = 0.006, Prolene vs PDS NS

**Fig. 3:**
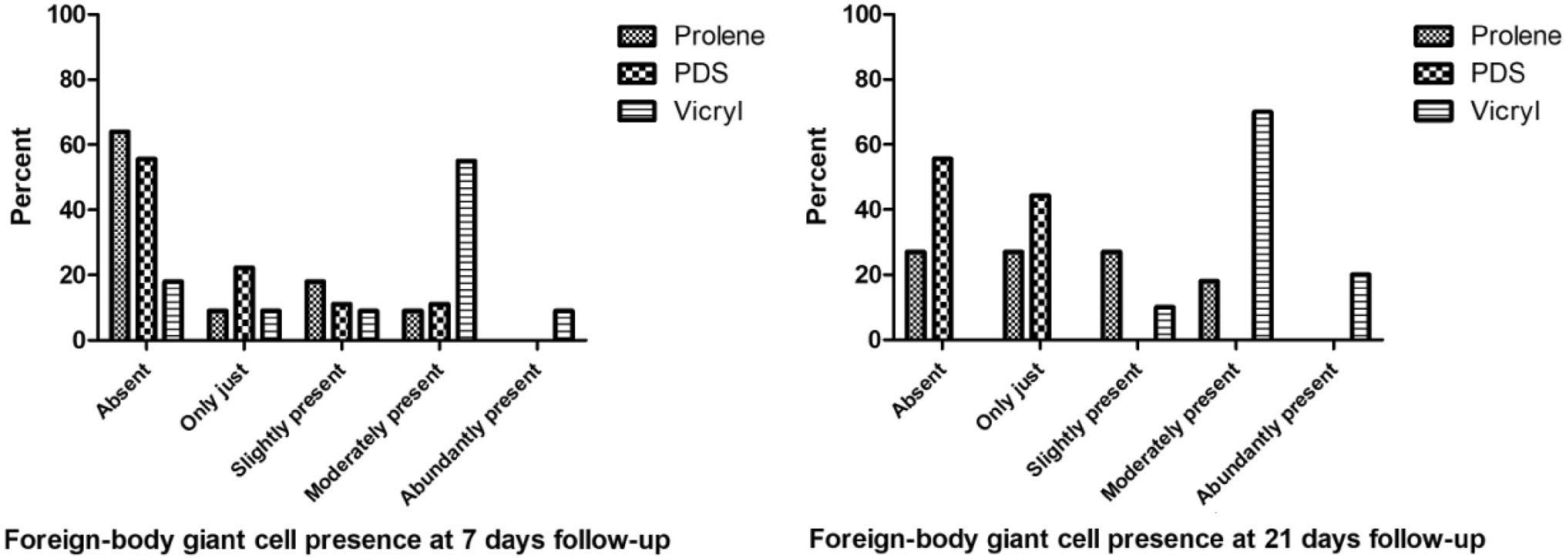
Semi-quantitative scoring of the presence of foreign body giant cells at 7 days and 21 days follow-up per suture type. At 7 days: Vicryl vs Prolene *p* = 0.010, Vicryl vs PDS NS, Prolene vs PDS NS; at 21 days: Vicryl vs Prolene *p* = 0.001, Vicryl vs PDS *p* < 0.001

The presence of granulocytes ranged from absent to abundantly present at 7 days of follow-up (Fig. 1a). The presence of granulocytes declined at 21 days of follow-up to a score of slightly present (Fig. 1b). At 7 days and 21 days, the differences between the three groups were not significant, *p* = 0.621 and *p* = 0.539, respectively. Macrophages were slightly to abundantly present at seven days of follow-up, without significant differences between the groups (*p* = 0.257). The overall presence of macrophages decreased but remained significantly higher in the Vicryl group compared to both Prolene (*p* = 0.003) and PDS (*p* = 0.006) at 21 days of follow-up. The presence of macrophages did not differ significantly between Prolene and PDS after 21 days (*p* = 0.324) (Fig. 2). The Vicryl group showed the highest presence of foreign body giant cells at both time points compared to both Prolene (*p* = 0.010) at both 7 days and 21 days (*p* = 0.001) and compared to PDS at 21 days (*p* < 0.001). At 7 days follow-up, no significant difference was detected between Vicryl and PDS regarding foreign body giant cells (*p* = 0.021). Furthermore, Prolene and PDS were comparable regarding the presence of foreign body giant cells at both time points (*p* = 0.830 and 0.053, respectively) (Fig. 3).

**Fig. 4:**
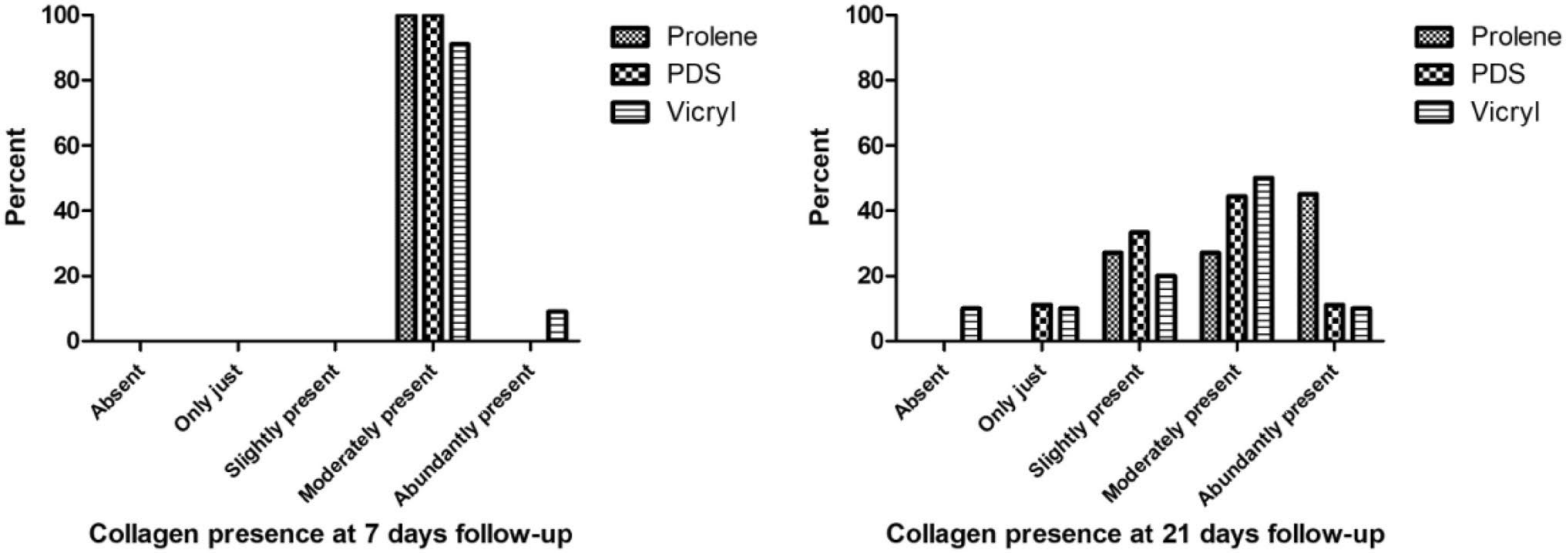
Semi-quantitative scoring of the presence of collagen at 7 days and 21 days follow-up per suture type. At 7 days: *p* = 0.403, at 21 days: *p* = 0.202

Collagen was moderately present at 7 days in almost all samples (*p* = 0.403). After 21 days, no significant difference was detected between the three groups regarding the presence of collagen (*p* = 0.202) (Fig. 4).

### qPCR

No samples were excluded in the gene expression analysis. Expression of genes in the PDS group was set as the norm and the expression in the Prolene and Vicryl groups was presented in relation to set norm.

#### Macrophage polarisation

**Fig. 5:**
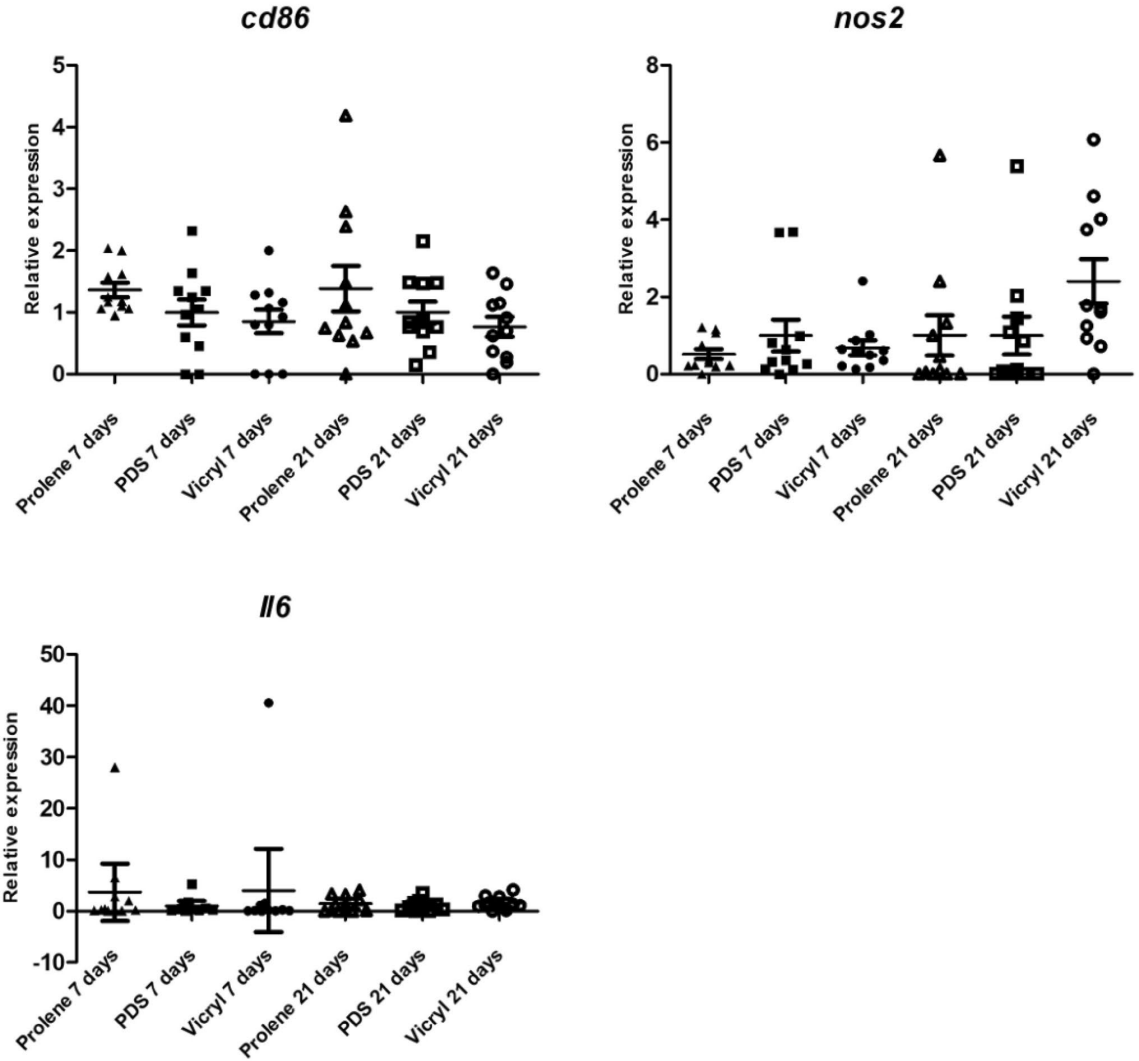
Scatter plots of relative expression of genes typically expressed by macrophage subtype 1 *(cd86, nos2 and il6)* at 7 days and 21 days follow-up. The expression in the Vicryl and Prolene groups is depicted in relation to the PDS group, which was set as the norm

Two macrophage subtypes are known, subtype 1 (M1) has pro-inflammatory properties and is associated with tissue injury. Subtype 2 (M2) is associated with extracellular matrix remodelling and regulation of fibroblasts [34–36]. To evaluate the presence of macrophage subtype 1, the expression of interleukin 6 (*il6*), *nos2* and *cd86* was determined. *Nos2* expression was a 2-fold lower in the Prolene group at 7 days follow-up, compared with the PDS group, but not significantly (*p* = 0.932). *Il6*, *cd86* and *nos2* expression did not differ significantly between groups on both time points (see Fig. 5).

**Fig. 6:**
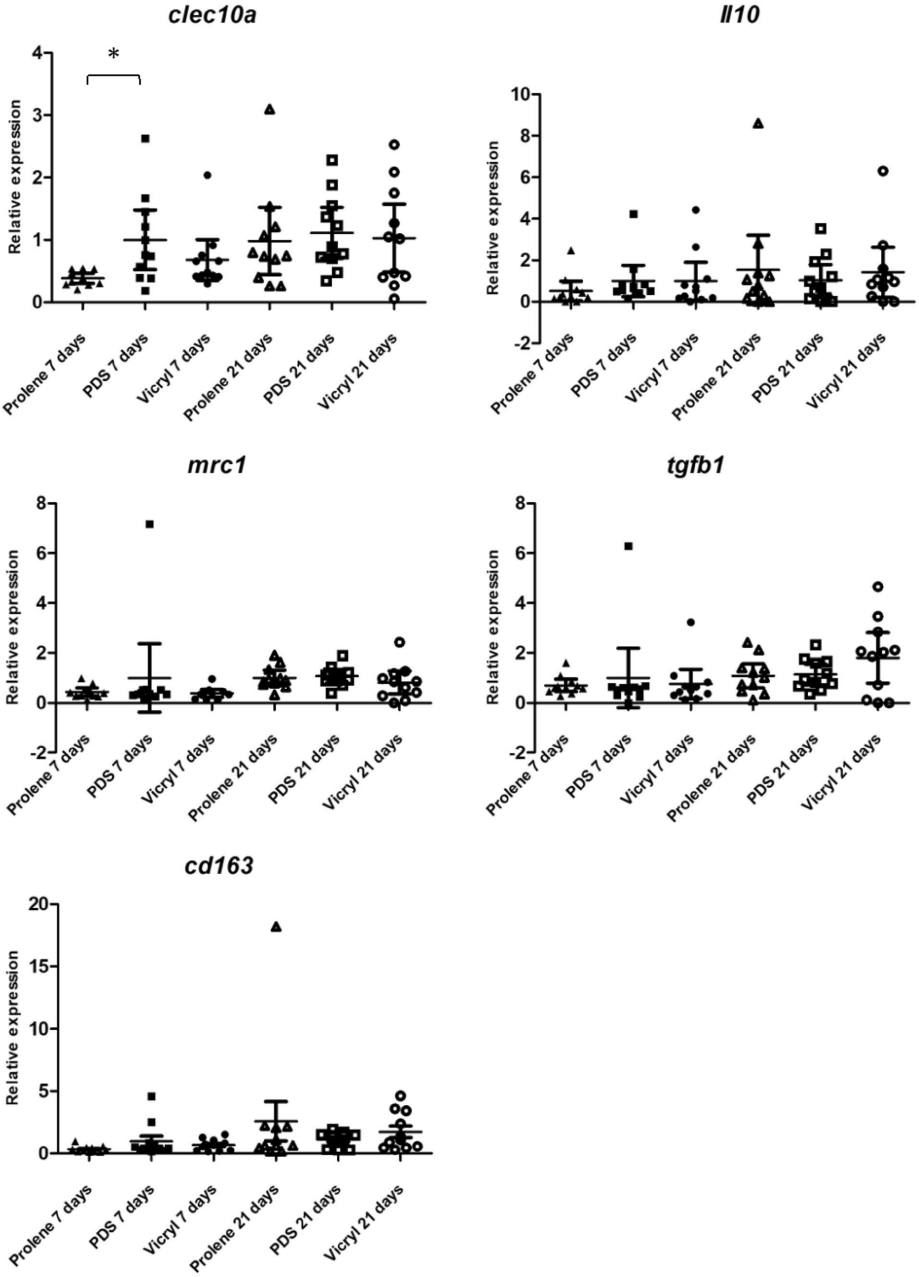
Scatter plots of relative expression of genes typically expressed by macrophage subtype 2 *(clec10a, il10, mrc1, tgfb1, cd163)* at 7 days and 21 days follow-up. The expression in the Vicryl and Prolene groups is depicted in relation to the PDS group, which was set as the norm. *p = 0.014

M2 polarisation is induced by interleukin 10 (*il10*) [36, 37] and macrophage subtype 2 is identified by an increased expression of *i10*, *clec10a*, *mrc1* (cd206), *cd163* and *tgfb1*. *Tgfb1* is typically expressed by the macrophage subtype 2c [36, 38–40]. A significantly higher expression of *clec10a* was found in the PDS group in comparison to Prolene at 7 days follow-up, namely 2.5-fold higher (*p* = 0.007). At 21 days, no significant differences were detected and the *clec10a* expression in both the Prolene and Vicryl groups was comparable to the PDS group. Although not significantly, a twofold lower *il10* expression was observed at 7 days follow-up in the Prolene group compared to the PDS group. *Mrc1* expression was 2.5-fold higher in the PDS group compared to Prolene and Vicryl at 7 days follow-up (*p* = 0.761) without reaching significance. The expression of *tgfb1* was a 1.4-fold lower in the Prolene group compared to the PDS group at 7 days follow-up (*p* = 0.619). A 3.3-fold and 1.4-fold lower expression of *cd163* was encountered in the Prolene and Vicryl groups, respectively, in comparison to the PDS group at 7 days follow-up (*p* = 0.117). The differences between groups in expression of *il10*, *mrc1*, *tgfb1* and *cd163* were not significant at both time points (see Fig. 6).

#### Vascularisation and fibroblast expression

**Fig. 7:**
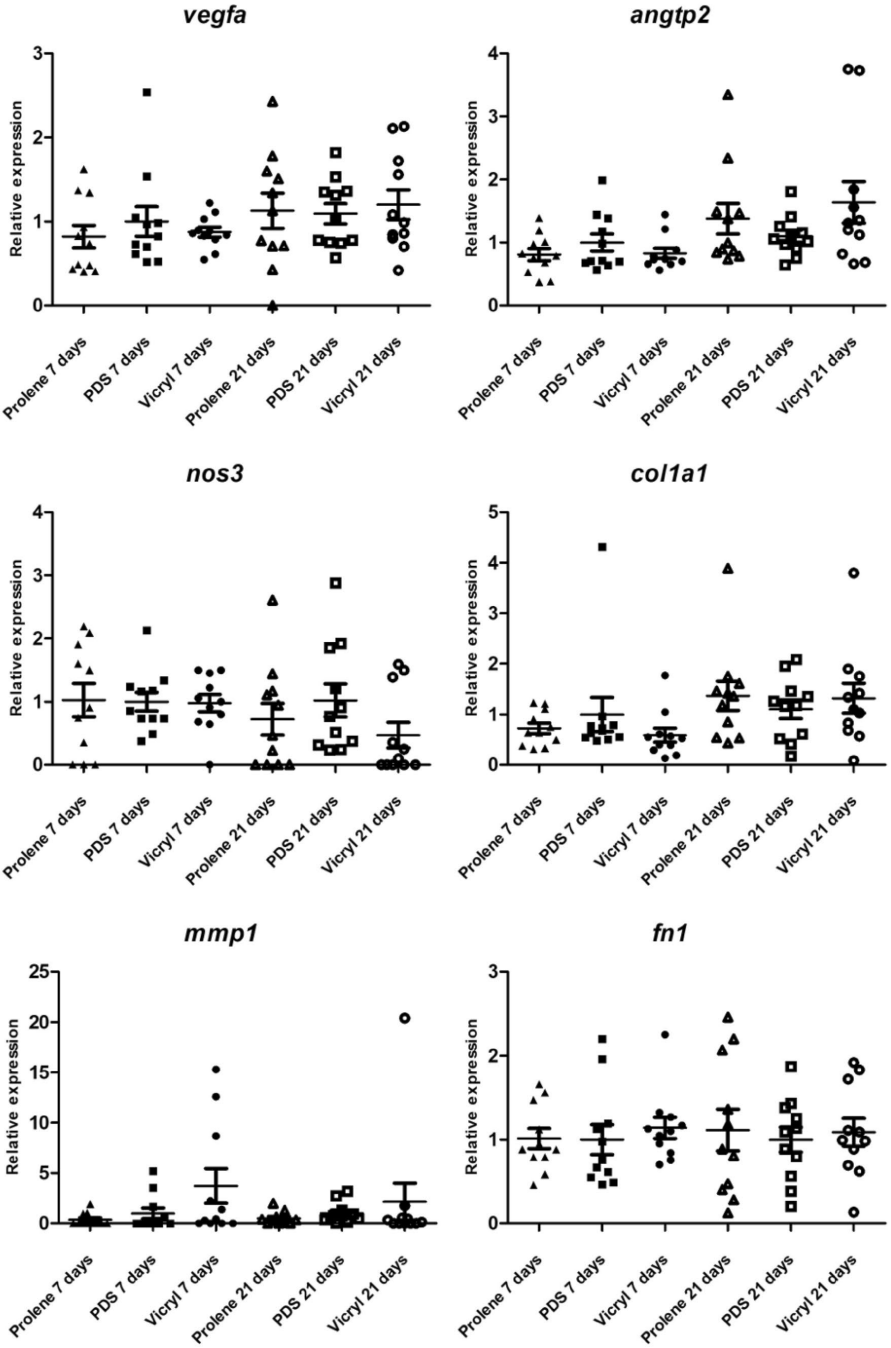
Scatter plots of relative expression of genes typically expressed in case of vascularisation (*vefga, angtp2 and nos3)* and by fibroblast *(col1a1, mmp1 and fn1)* at 7 days and 21 days follow-up. The expression in the Vicryl and Prolene groups is depicted in relation to the PDS group, which was set as the norm

Furthermore, vascularisation was evaluated using *vegfa*, *angtp2* and *nos3* expression [41, 42], but no significant difference was detected between the groups regarding the expression (see Fig. 7). Collagen type 1 alpha 1 chain (*col1a1*), *mmp1* and *fn1* expression were a measure for fibroblast activity [39, 43]. Prolene induced a 2.5-fold lower expression of *mmp1* at 7 days (*p* = 0.161) and twofold lower at 21 days (*p* = 0.180) in comparison to PDS. The comparison between groups regarding the expression of *fn1*, *col1a1* and *mmp1* did not reveal significant differences (see Fig. 7).

## Discussion

The technique for closure of the abdominal wall is evolving to prevent incisional hernia development. Both the use of a small bite technique shows a reduction in the incidence of incisional hernia [22, 23], as well as mesh placement after a laparotomy prevents incisional hernia in high-risk patients [6, 18]. Reports in the literature regarding the optimal suture material for the closure of the abdominal wall are conflicting and focus on clinical outcomes, such as incisional hernia, suture sinuses and wound pain [4, 21, 25, 44].

The meta-analyses comparing non-absorbable sutures versus slow-absorbable sutures are confounded by factors in the study population; multiple types of incisions, emergency versus elective surgery and different suture materials or techniques are included. In the comparison between non-absorbable and slow-absorbable sutures, no differences are detected regarding the incisional hernia rate [21, 25]. However, slow-absorbable sutures do result in less wound pain and suture sinuses. This experiment intends to provide a pathophysiological foundation for the choice in suture material, hypothesising that improved healing of the abdominal wall leads to a reduction of the incidence of incisional hernia.

A fast-absorbable, slow-absorbable and non-absorbable suture material was compared in a rat model. In general, the foreign body reaction to biomaterials is subdivided into four phases: protein absorption, cell recruitment and adhesion, foreign body giant cell formation, and finally extracellular matrix formation and fibrotic encapsulation [45, 46]. In this experiment, the focus lies on the last three phases. Tissues were microscopically examined regarding the presence of different types of cells and collagen fibres. Consequently, the gene expression was determined using qPCR to explore vascularisation, fibroblast activity and macrophage polarisation.

The presence of macrophages was significantly higher in the Vicryl group after 21 days and simultaneously more foreign body giant cells were encountered compared to Prolene at 7 days and compared to PDS at 21 days. PDS and Vicryl are both absorbable sutures which rely on hydrolysis for degradation [47, 48]. Polylactic and polyglycolid acids, polymers both present in Vicryl, depend on phagocytosis by macrophages and especially foreign body giant cells for complete absorption [45, 49]. Vicryl elicits a stronger macrophage response in comparison to Prolene and PDS, which also results in more foreign body giant cells. The interaction between host and suture might play a role in its degradation and absorption. Although, in that case a similar finding would be expected in PDS. PDS differs from Vicryl in the polymer used and the fact that it is a monofilament suture in contrast to Vicryl which is braided [48]. These factors contribute to the difference in absorption rate between PDS and Vicryl, which, in the case of the faster absorption rate of Vicryl, can negatively affect the occurrence of incisional hernia. This concurs with clinically based results from a previous report, in which slow-absorbable sutures were considered superior regarding the incidence of incisional hernia compared to fast-absorbable sutures [20].

After determining the quantity of macrophages present, the polarisation between macrophage subtype 1 and 2 was evaluated using qPCR. Macrophage subtype 2 and its signalling play an essential role in liver regeneration, skeletal muscle healing and scar formation after injury of the skin [50–52]. Therefore, it was hypothesised that the macrophage subtype 2, which modulates extracellular matrix and activates fibroblasts [34–36], would have a positive effect on abdominal wall healing.

C*lec10a* expression, typical for macrophage subtype 2, was significantly higher in the PDS group compared to the Prolene group. This suggests a more dominant presence of macrophage subtype 2 in the healing process. A striking number of genes (*il10*, *mrc1*, *cd163*, *tgf*-*b*) typical for subtype 2 macrophages showed higher expression, although not significantly, when the abdominal wall was closed with PDS instead of Prolene after 7 days follow-up. The expression levels of the genes *mrc1* and *cd163* were higher in the presence of PDS compared to Vicryl and Prolene after 7 days, without reaching significance. *Nos2*, typically expressed by the macrophage subtype 1, and *mmp1* showed a similar pattern. Although no significant differences could be detected, multiple genes typically expressed by subtype 2 macrophages were high in expression in the PDS group versus one subtype 1 specific gene. This could be interpreted as a distinct pattern, suggesting a dominant macrophage subtype 2 presence after abdominal wall closure with PDS. The effect disappears after 21 days of follow-up, which coincides with the progression of the healing process. The findings regarding macrophage polarisation concur with reports on scaffolds of different biomaterials, concluding a mainly anti-inflammatory macrophage polarisation in reaction to PDS (polydioxanone). Regarding polypropylene and polylactic acid, both the pro-inflammatory and anti-inflammatory macrophage responses were reported [53].

The presence of different cell types in the analysed samples might result in various expression patterns, which could influence qPCR results. In addition, the macrophage polarisation is described as a spectrum rather than a black and white differentiation [54–56]. This may cause heterogeneity in the qPCR data and might limit the ability to reach a statistical difference. This might also be an argument for a larger sample size than calculated for this experiment to reach adequate power.

Recording the mere presence of macrophages no longer suffices in evaluation of the healing process of the abdominal wall and novel insights in macrophage polarisation need to be taken into account. Subtyping and activation of macrophages provide additional information on the regenerative process taking place in the abdominal wall. The anti-inflammatory subtype 2 macrophage is associated with tissue regeneration and is, therefore, assumed to have a positive effect on the healing process [34–36, 50–52]. The results of this experiment suggest a favourable macrophage response to PDS in comparison to Prolene and Vicryl, which in turn might benefit the regenerative capacity of the abdominal wall. This provides a new perspective on the dilemma of appropriate suture material in abdominal wall closure, which is of added value to the existing literature, which is mostly based on clinical outcomes.

## Conclusion

This study provides an argument carefully supporting the use of PDS suture, in comparison to Vicryl or Prolene for closure of the abdominal wall. This is based on limited evidence indicating macrophage subtype 2 polarisation, a favourable macrophage response which could play a role in the regeneration process of the abdominal wall.

## Electronic supplementary material

Below is the link to the electronic supplementary material.

Supplementary material 1 (DOCX 19 kb)
