# Supplementary material for: Prevention of incisional hernia using different suture materials for closing the abdominal wall: a comparison of PDS, Vicryl and Prolene in a rat model
Source: Hernia. 2019 May 20;24(1):67–78. doi: 10.1007/s10029-019-01941-9 (PMC7007910; doi:10.1007/s10029-019-01941-9)
Supplement: Supplementary file 1 — Supplementary material 1 (DOCX 19 kb) [file 10029_2019_1941_MOESM1_ESM.docx]

| **Gene name** | **Product length** |  | **GC%** | **sequence** |
| --- | --- | --- | --- | --- |
| *rplp0* (ribosomal protein lateral stalk subunit P0) | 190 | f | 55.00 | CCTCACCGAGATTAGGGACA |
|  |  | r | 45.00 | ATCGCTCAGGATTTCAATGG |
| *actb* (actin, beta) | 297 | f | 55.00 | CCGCGAGTACAACCTTCTTG |
|  |  | r | 55.00 | CAGTTGGTGACAATGCCGTG |
| *il6* (Interleukin 6) | 246 | f | 57.14 | CTCTCCGCAAGAGACTTCCAG |
|  |  | r | 47.62 | TTCTGACAGTGCATCATCGCT |
| *nos2* (iNOS) | 234 | f | 52.38 | TAGTCAACTACAAGCCCCACG |
|  |  | r | 60 | GTGAGGAACTGGGGGAAACC |
| *cd86* (CD86) | 164 | f | 45.45 | AGACATGTGTAACCTGCACCAT |
|  |  | r | 55 | TACGAGCTCACTCGGGCTTA |
| *il10* (Interleukin 10) | 186 | f | 52.38 | CGACGCTGTCATCGATTTCTC |
|  |  | r | 60.00 | CAGTAGATGCCGGGTGGTTC |
| *clec10a* (C-type lectin domain containing 10a) | 164 | f | 60.00 | GAGGCTTGAGCCAGAAGGTG |
|  |  | r | 52.38 | TGCTGAGCCGTTGTTCTTGAG |
| *mrc1* (mannose receptor C typ 1) | 212 | f | 60.00 | CCCGCTCCTCAAGACAATCC |
|  |  | r | 55.00 | AAATACGGTGACTGCCCACC |
| *cd163* (CD163) | 131 | f | 60 | CTCTGAAGCGACGACAGACC |
|  |  | r | 50 | ATGCCAACCCGAGGATTTCA |
| *tgfb1* (transforming growth factor-β) | 115 | f | 60.00 | GGCTGAACCAAGGAGACGGA |
|  |  | r | 55.00 | CCTCGACGTTTGGGACTGAT |
| *vegfa* (vascular endothelial growth factor a) | 235 | f | 60 | AGAAGGGGAGCAGAAAGCCC |
|  |  | r | 47.83 | GATCCGCATGATCTGCATAGTGA |
| *angpt2* (angiopoietin 2) | 168 | f | 55 | CATGATGTCATCGCCCGACT |
|  |  | r | 52.38 | TCCATGTCACAGTAGGCCTTG |
| *nos3* (eNOS) | 139 | f | 52.38 | GAATGGAGAGAGCTTTGCAGC |
|  |  | r | 60 | CCGCCAAGAGGATACCAGTG |
| *col1a1* (collagen type 1 alpha 1 chain) | 237 | f | 60 | CTGACTGGAAGAGCGGAGAG |
|  |  | r | 55.00 | CAGGATCGGAACCTTCGCTT |
| *mmp1* (matrix metallopeptidase 1) | 144 | f | 55.00 | AAGGCCACTGGTGATCTTGC |
|  |  | r | 43.48 | GGTATTTCCAGACTGTTTCCACA |
| *fn1* (Fibronectin 1) | 165 | f | 63.16 | TCCCCTCCCAGAGAAGTGG |
|  |  | r | 43.48 | TTGGGGAAGCTCATCTGTCTTTT |

Supplementary Table 1 gene specific primers used, build using a primer designing tool (Primer-blast) (1).

1. Ye J, Coulouris G, Zaretskaya I, Cutcutache I, Rozen S, Madden TL. Primer-BLAST: a tool to design target-specific primers for polymerase chain reaction. BMC Bioinformatics. 2012;13:134.
